# Lengthening of knee flexor muscles by percutaneous needle tenotomy: Description of the technique and preliminary results

**DOI:** 10.1371/journal.pone.0182062

**Published:** 2017-11-07

**Authors:** Alexis Schnitzler, François Genêt, Aurélie Diebold, Laurence Mailhan, Claire Jourdan, Philippe Denormandie

**Affiliations:** 1 Department of Physical Medicine and Rehabilitation, CHU R.Poincaré, Garches, France; 2 Department of orthopaedic surgery, CHU R.Poincaré, Garches, France; Georgia Regents University, UNITED STATES

## Abstract

**Background:**

Knee flexion contractures occur frequently in non-ambulatory, aged persons and persons with central nervous system lesions, rendering positioning and nursing care difficult. There are often risks associated with surgical interventions.

**Objective:**

To evaluate the effectiveness of percutaneous needle tenotomy to lengthen the knee flexor muscles and improve passive function.

**Methods:**

This was a retrospective study of all patients who underwent percutaneous needle tenotomy between 2012 and 2014. Tenotomy was carried out in the semi-tendinosus, biceps femoris and gracillis muscles under local anesthesia. The procedure took no more than 40 minutes. Range of motion (ROM) was evaluated immediately post-operatively and 3 months later.

**Results:**

Thirty-four needle tenotomies were carried out. Mean lack of knee extension was 94.2° (range 35–120°) pre-op, (range 15–90°; p<0.05) immediately post-op and 50.1° (range 10–90°; p<0.05) three months later, thus a mean increase of 44.1° knee extension (range 0–90°). All care and positioning objectives were achieved. There were no complications and procedure-related pain was rated as 3-4/ 10.

**Conclusions:**

Needle tenotomy was well tolerated and yielded a significant increase in ROM with no unwanted effects. All objectives were achieved. This technique could be used in an ambulatory care setting or within institutions for severely disabled individuals.

## Introduction

Muscle contractures are common following acquired (such as stroke or brain injury) or degenerative (such as dementia or Parkinson’s disease) lesions of the central nervous system [1,2]. The prevalence of muscle contractures has been particularly studied in aged people, showing that 22% to 55% of nursing home residents have contractures with a major loss of joint range of motion (ROM) [3,4].

Contractures of the hamstring and gracilis muscles can significantly reduce function. This is well documented in ambulant patients with cerebral palsy [5] however, there are few studies of the prevalence and treatment of contractures in non-ambulant patients such as those in a minimally conscious (MCS) or persistent vegetative (PVS) state, or severely impaired or fragile older patients. One transversal study reported a 75% prevalence of contractures of the knee flexor muscles, defined as a loss of at least 5° of passive knee extension, in nursing home residents [6]. Contractures of the knee flexor muscles in aged, fragile patients can have a severe impact on their functional capacity. Acquired deforming hypertonia makes nursing care difficult in 59% of cases and positioning in a chair uncomfortable or impossible in 49% of cases [4]. Martin et al. showed that surgical lengthening of the knee flexors combined with the use of an external fixator yielded excellent results in 57 patients [7]. However, the use of general anesthesia and the high level of post-operative care necessary can be risky in fragile patients. Aponeurotomy, first described by Lemursiaux to improve joint ROM in patients with Dupyutren’s contractures of the hand [8,9], is a simple, relatively painless technique with few side effects. It constituted a real progress for the treatment of this condition. Since then, several teams have used this technique for the treatment of tendinitis [10,11] and found it to be safe and effective, leading it to become more widespread.

We have carried out two previous studies, one in cadavers and the other on fixed flexion deformities in elderly institutionalized persons, which showed that the technique was safe and effective [12, 13].

The aim of this retrospective study was thus to evaluate a technique of percutaneous needle tenotomy developed in our multidisciplinary neuro-orthopedic department to lengthen the semitendinosus, biceps femoris and gracillis muscles. This technique has the advantage of avoiding the risks of surgery and anesthesia in aged or frail patients. This study reports on the technique and the first results obtained.

## Methods

The study was retrospective and included all the patients who underwent lengthening of the knee flexor muscles by needle tenotomy in our multidisciplinary neuro-orthopaedic department from 2012 to 2014. Patients are referred to the department if they are elderly or have disabling muscle contractures related to a neurological condition. All the patients had severe contractures of the knee flexor muscles and reduced function as a result. Loss of functional capacity was reported by health-care teams or the patients’ families. The patients all had contraindications to usual treatments for contractures (such as botulinum toxin injections) and they were considered by their general practitioners to be too fragile to undergo general anaesthesia and classic surgery. Moreover, the frequent presence of behavioral disorders made hospitalization complicated. The aim of the treatment was to rapidly improve patients on the day of their first consultation. The management of each patient was determined by a physiatrist and an orthopedic surgeon.

Our ethics committee confirmed that retrospective studies in France do not require ethical approval. The Jardé law considers that research on retrospective data is excluded from this framework http://social-sante.gouv.fr/systeme-de-sante-et-medico-social/recherche-et-innovation/article/recherches-impliquant-la-personne-humaine. Equally, permission from the CNIL was not required: https://www.cnil.fr/fr/declaration/mr-003-recherches-dans-le-domaine-de-la-sante-sans-recueil-du-consentement and a retrospective waiver was received form the CNIL for the study. Data were anonymized prior to analysis. Two authors (PD and AS) were directly responsible for patient care or the decision to perform percutaneous needle tenotomy on the patients.

### Surgical procedure

Tendons were located by palpation of the sub-cutaneous cords of the hamstring and the gracilis muscles during passive knee extension. Since the muscles were shortened, palpation of the semitendinosus, gracilis and biceps femoris tendons was straight forward. Palpation of the semimembranosus tendon was more difficult because of its deeper, wider insertion.

The site to be injected was prepared with Povidone-iodine. Next, the skin and subcutaneous tissues were infiltrated with a total dose of 10 mL of 1% Lidocaine using a hypodermic needle. The point of injection was 5 cm proximal to the distal insertion of the semitendinosus, gracilis and biceps femoris muscles. Anesthetic solution was also injected directly into the tendon. Once adequate anesthesia was achieved, another needle (gauge = 16.5, 1.6 x40 mm, classically used for paracentesis) was used to repeatedly fenestrate the tendon of the three muscles. The needle was inserted perpendicularly to the tendon, not more than 1cm deep (to avoid injury to the deeper neuro-vascular structures). As a general rule, one puncture wound was necessary for each tendon. Initial passes through the tendon were typically met with resistance, producing palpable and audible crepitation. As the needle was passed repeatedly, the tissues softened, and crepitation reduced. An extension force was manually applied on the lower limb throughout the whole procedure in order to tension the tendon. The total duration of the intervention was 30–40 minutes for all three tendons. The intervention was considered to be completed once the sub-cutaneous cord disappeared and knee ROM increased.

All patients resided in nursing homes and received only a small amount of physiotherapy (less than 1 hour per week), thus no specific rehabilitation was carried out following the tenotomy. Advice was simply given to continue to stretch the treated muscles and to ensure that the patient was regularly positioned in a chair.

The demographic characteristics (age, diagnosis, side of treatment) of the patients included in the study were recorded.

### Assessments

The primary aim was to increase passive knee extension. Measurement of passive knee extension was carried out using a goniometer, before, immediately after the intervention and 3 months later. These measurements were mostly taken with the hip flexed at 45°, however this depended on the mobility of the hip joint. The pre- and post-operative measurements were carried out by the same operator who attempted to standardize the hip position for each patient.

The secondary aim was to improve functional capacity. One single aim was determined by consensus with the patients’ health-care teams and, when possible, their families. Improvement was evaluated by carers during the 3 month follow up consultation by a closed ‘yes’ or ‘no’ question. For patients who had sufficient cognitive capacity, pain was recorded after the procedure using a visual analogue scale. Any unwanted effects were also evaluated during the 3 month follow up.

### Statistical analysis

Data are reported as means, standard deviations (SD), ranges and quartile percentages. A Wilcoxon test was used to compare normally distributed quantitative variables. All *p* values were two-tailed, and a *p* value of <0.05 was considered statistically significant.

## Results

Thirty-four percutaneous needle tenotomies were carried out during the study period. Eight patients underwent bilateral interventions. Average age was 75 years. The reasons for the needle tenotomies were: major difficulty or impossibility to position the patient in bed or a wheelchair (20 patients), difficultly to carry out nursing care (4 patients) and to facilitate gait (2 patients).The mean lack of knee flexion was 94.2° (range 35–120°) prior to the intervention, 51.3° (range 15–90°; p<0.05) immediately after and 50.1° (range 10–90°; p<0.05) 3 months later. The average increase in knee extension at 3 months was 44.1° (range 0–90°).

No unwanted effects occurred during or immediately following the interventions. A simple plaster was sufficient to cover the skin wounds, no stitches were required. No patients experienced much pain during the intervention.

Three patients (1, 5 and 8, all bilateral tenotomies) were able to evaluate procedure-related pain. Their ratings were respectively 3/10, 3/10 and 4/10. No cutaneous, infectious, hemorrhagic or neurological complications were reported either immediately post-operatively or at the 3 month follow up.

## Discussion

This pilot study showed that a simple technique to lengthen the semi-tendinosus, biceps femoris and gracilis muscles was effective and well tolerated. There was a large increase in ROM following the intervention which was maintained at 3 months. Nursing care was facilitated and all patients could be positioned in a chair. This technique is rapid, safe and relatively simple to carry out.

Open surgical lengthening of muscle contractures can produce greater increases in ROM than the results found in the present study. A previous study showed an increase of 62° of knee extension in patients in who had problems with positioning (MCS paraplegia and tetraplegia) [7], compared with a mean increase of 44° in the present study. However the complexity of the intervention, the general anesthesia and sometimes the use of an external fixator for several weeks make the risks (bed-sores, for example) too high for older patients. Moreover, the increase in ROM in the present study was sufficient to achieve comfortable positioning of all the patients and to reduce pressure points. We therefore propose that this technique should be an option for fragile patients and that more complex interventions should be reserved for patients for whom the aims are more ambitious.

Percutaneous surgery is another alternative to open surgery. It is similar to the technique described in the present article but usually requires general anesthesia [14, 15,16]. Although the intervention involves a small incision, healing can be difficult because of the tension placed on the skin. This is not a problem following needle tenotomy, and no stitches are required since the needles used (16 G) result in a small-diameter puncture wound.

Needle tenotomy therefore appears to be a promising technique. Other studies have evaluated its use for the treatment of tennis elbow, showing similar results to studies of percutaneous techniques with no particular unwanted effects [11,17,18]. Needle tenotomy of the triceps sural tendon, combined with serial casting for equinus foot in children [19] has also yielded good results with excellent toleration of the intervention. Moreover, the risk of vascular or neurological complications, infections, cutaneous problems or bed sores is reduced because the reduction of the deformity is incomplete and there is no post-operative immobilization. There is, however a potential risk of injuring neighboring nerves and vessels during needle tenotomy. This risk is, however, quite small since it is generally easy to locate the tendons, and other important structures (such as the fibular nerve and the femoral artery) are usually located several centimeters away from the puncture sites. Because, the semi-membranousus muscle is not included in the intervention, deep punctures that could involve greater risks are not necessary. However, when the aim is to achieve more complete knee extension, this muscle can be treated using the same technique. In the future, the technique could be developed to improve mobility at other joints (hip flexion deformity, equinovarus etc.) with more ambitious objectives (e.g. standing and/or gait). Ultrasound could be useful to visualize neighboring structures and thus avoid them [20], however we did not use ultrasound because the aim was to develop a simple technique. Indeed, the technique described in the present study could be carried out within the centers or institutions in which the patients are cared for.

### Study limitations

The study was retrospective and the evaluators were not blinded. However the increases in ROM were substantial and thus the lack of blinding probably had a very small impact on the results.

A 3 month follow up is not sufficient to know if the improvements were maintained over a long period of time. However, some patients were reviewed more than one year after the intervention and were found to have maintained the ROM. This suggests that the likelihood of recurrence of the contracture is small (data not presented).

Pain was not evaluated for most patients. However, none of the patients who were able to express themselves reported excessive pain during the intervention. Equally, none of the carers accompanying non-communicating patients noticed signs of excess pain during the intervention.

## Conclusion

Patients who had not been able to sit in a chair for several years, and who could not undergo surgery because of the risks, were able to be positioned in a chair following the intervention presented here. The avoidance of long-term bed rest for older or frail persons has medical benefits (cardio-pulmonary, cutaneous, constipation, bony etc.) and improves quality of life.

This technique could be used in an ambulatory care setting or within institutions for severely disabled individuals, following training with an experienced orthopedic surgeon. It could also be used for other muscles (biceps brachialis, gastrocnemius, finger and toe flexors etc.).

## Supporting information

S1 File All the data for the article can be found in: Data file.docx. (DOCX)
