# Supplementary material for: Lengthening of knee flexor muscles by percutaneous needle tenotomy: Description of the technique and preliminary results
Source: PLoS One. 2017 Nov 7;12(11):e0182062. doi: 10.1371/journal.pone.0182062 (PMC5675412; doi:10.1371/journal.pone.0182062)
Supplement: S1 File — (DOCX) [file pone.0182062.s001.docx]

S1 file

Table1: Patient characteristics and results of the needle tenotomy of the biceps femoris, gracilis and semitendinosus muscles.

|  | | | | | | | | | | |
| --- | --- | --- | --- | --- | --- | --- | --- | --- | --- | --- |
| **Patient no.** | **Gender** | **Age (years)** | **Main pathology** | **Side of intervention** | **Pre knee ext angle (°)** | **Associated interventions** | **Post knee ext angle (°)** | **Knee ext angle at 3 months (°)** | **Change in ext T0-3 months (°)** | **Aim of intervention** |
| *1* | F | 72 | Dementia | R | 100 | Phenol AL | 45 | 40 | +60 | Positioning in W/C |
| *1* | F | 72 | Dementia | L | 100 | Phenol AL | 45 | 40 | +60 | Positioning in W/C |
| *2* | F | 97 | Dementia | R | 120 | Phenol AL | 75 | 80 | +40 | Nursing care |
| *3* | F | 94 | Dementia | R | 100 | Phenol AL | 80 | 80 | +20 | Nursing care |
| *4* | F | 79 | Dementia + TBI | L | 90 | 0 | 60 | 60 | +30 | Positioning in W/C |
| *4* | F | 79 | Dementia + TBI | R | 100 | 0 | 60 | 60 | +40 | Positioning in W/C |
| *5* | M | 68 | CP | R | 70 | BTI SM | 20 | 30 | +40 | Positioning in W/C |
| *5* | M | 68 | CP | L | 50 | BTI SM | 20 | 30 | +20 | Positioning in W/C |
| *6* | M | 70 | Stroke | R | 110 | 0 | 80 | 70 | +40 | Positioning in W/C |
| *7* | F | 97 | Dementia | R | 120 | Phenol AL | 70 | 70 | +50 | Nursing care |
| *8* | M | 61 | Stroke | L | 90 | 0 | 20 | 30 | +60 | Positioning in W/C |
| *8* | M | 61 | Stroke | R | 90 | 0 | 20 | 30 | +60 | Positioning in W/C |
| *9* | F | 85 | Dementia | L | 100 | 0 | 20 | 20 | +80 | Positioning in W/C |
| *10* | M | 75 | Stroke | R | 90 | 0 | 45 | 30 | +60 | Positioning in W/C |
| *11* | F | 90 | Dementia + PD | L | 110 | 0 | 20 | 30 | +80 | Nursing care |
| *12* | M | 80 | Stroke | R | 110 | 0 | 80 | 80 | +30 | Positioning in W/C |
| *13* | M | 86 | PD | R | 50 | 0 | 30 | 20 | +30 | Facilitation of gait |
| *13* | M | 86 | PD | L | 50 | 0 | 30 | 20 | +30 | Facilitation of gait |
| *14* | M | 59 | MS | R | 100 | Phenol AL | 50 | 40 | +60 | Positioning in W/C |
| *14* | M | 59 | MS | L | 90 | Phenol AL | 50 | 40 | +50 | Positioning in W/C |
| *15* | M | 89 | Stroke | L | 120 | 0 | 70 | 75 | +45 | Positioning in bed and W/C |
| *15* | M | 89 | Stroke | R | 120 | 0 | 70 | 85 | +35 | Positioning in bed and W/C |
| *16* | F | 86 | Dementia | R | 100 | 0 | 70 | 80 | +20 | Positioning in W/C |
| *17* | F | 90 | Dementia | R | 110 | 0 | 40 | 40 | +70 | Positioning in W/C |
| *18* | F | 27 | CP | L | 90 | 0 | 70 | 90 | 0 | Positioning in W/C |
| *19* | M | 32 | Anoxia | R | 100 | Tenotomy AL | 40 | 10 | +90 | Positioning in bed and W/C |
| *20* | M | 73 | Stroke | L | 35 | 0 | 15 | 15 | +20 | Facilitation of gait |
| *21* | M | 57 | PD | R | 90 | Tenotomy AL + GM | 40 | 40 | +50 | Positioning in bed and W/C |
| *22* | F | 90 | Stroke | L | 90 | Tenotomy GM | 30 | 30 | +60 | Positioning in W/C |
| *23* | M | 45 | MS | R | 110 | Tenotomy sartorius + GM + phenol AL | 80 | 80 | +30 | Positioning in bed and W/C |
| *24* | F | 92 | Alzheimer | L | 100 | 0 | 80 | 80 | +20 | Positioning in W/C |
| *24* | F | 92 | Alzheimer | R | 100 | 0 | 80 | 80 | +20 | Positioning in W/C |
| *25* | F | 69 | Stroke | R | 120 | 0 | 90 | 70 | +50 | Positioning in W/C |
| *26* | F | 80 | Stroke | L | 80 | 0 | 50 | 30 | +50 | Positioning in W/C |
|  |  |  |  |  |  |  |  |  |  |  |
| *Mean* |  | 75 |  |  | 94 |  | 51 | 50 | +44 |  |
| *Range* |  | 27-97 |  |  | 35-120 |  | 15-90 | 10-90 | 0-90 |  |
|  | | | | | | | | | | |

Ext=extension, F= female, M=male, TBI= traumatic brain injury, CP= cerebral palsy, MS=multiple sclerosis, PD=Parkinson’s disease, phenol = intramuscular phenol injection, BTI= botulinum toxin injection, SM=semimembranosus AL= adductor longus, GM= gluteus medius, W/C=wheelchair
